# When and How Should Chinese Pregnant Women Exercise? A Longitudinal Study in China

**DOI:** 10.3390/ijerph17010180

**Published:** 2019-12-25

**Authors:** Mi Xiang, Masayuki Konishi, Huanhuan Hu, Mio Nishimaki, Hyeon-Ki Kim, Hiroki Tabata, Hisao Shimizu, Yue Fang, Xueyuan Li, Jiawei Xu, Zhiruo Zhang, Huigang Liang, Takashi Arao, Shizuo Sakamoto

**Affiliations:** 1 School of Public Health, Shanghai Jiao Tong University, Shanghai 200025, China; kou_mitsu@hotmail.com (M.X.); fangyue@sjtu.edu.cn (Y.F.); sharon1222@163.com (X.L.); xujiawei@sjtu.edu.cn (J.X.); 2 Faculty of Health Science, Tokoha University, Shizuoka 431-2102, Japan; m-konishi@hm.tokoha-u.ac.jp; 3 National Center for Global Health and Medicine, Tokyo 162-8655, Japan; hu.huanhuan@yahoo.com; 4 Japan Institute of Sports Sciences, Tokyo 115-0056, Japan; mionishimaki@fuji.waseda.jp; 5 School of Advanced Science and Engineering, Waseda University, Tokyo 162-8480, Japan; hk.kim@aoni.waseda.jp; 6 Faculty of Sport Sciences, Waseda University, Saitama 359-1192, Japan; tbt-aaa-0963@ruri.waseda.jp (H.T.); kouta-shun-ryo@akane.waseda.jp (H.S.); s.sakamoto@waseda.jp (S.S.); 7 Department of Business Information & Technology, Fogelman College of Business & Economics, University of Memphis, Memphis, TN 38152, USA; 8 Meiji Yasuda Life Foundation of Health and Welfare, Physical Fitness Research Institute, Tokyo 192-0001, Japan; tarao@waseda.jp

**Keywords:** physical activity, gestational weight gain, infant birthweight, Chinese women

## Abstract

This study aimed to examine when and how physical activity (PA) influences gestational weight gain (GWG) and infant birthweight (BW) by considering the PA’s total volume, timing, intensity, and type, controlling for the influence of energy intake. A total of 1272 participants in different stages of pregnancy were recruited from hospital. The associations between PA and GWG or BW in the latter half of pregnancy were significant. Women with the highest PA volume in the third trimester had significantly lower risks of inadequate and excessive GWG by 69% (OR = 0.31, 95%CI: 0.10–0.91) and 67% (OR = 0.33, 95% CI: 0.12–0.91), respectively, compared to women in the lowest quartile. Women who achieved the recommended moderate intensity of PA during their second and third trimesters, independent of total volume of PA, had infants with significantly lower BWs compared to those who did not (β = −0.15, SE = 66.33, *p* = 0.04; β = −0.20, SE = 64.54, *p* = 0.01, respectively). Therefore, the effects of total volume and intensity of PA on GWG and BW were different. Interventions to prevent inappropriate GWG and macrosomia may need to set different priorities and timing regarding total volume or intensity of PA.

## 1. Introduction

Inadequate or excessive gestational weight gain (GWG) is associated with adverse maternal and infant health. Inadequate GWG exposes women to increased risk of having preterm births, small-for-gestational age offspring, and neonatal morbidity, whereas women with excessive GWG are likely to suffer from gestational complications, postpartum weight retention, and macrosomia [1,2,3]. Birthweight (BW) is a common indicator of future adult health outcomes [4]. Macrosomia, defined as birth weight greater than 4 kg, is associated with adverse health consequences in adulthood, including adult obesity [5,6]. Although inappropriate GWG and high BW can increase risks for adverse outcomes for both mothers and children, more than half of pregnant women in China have inappropriate GWG [7] and the rate of macrosomia is still increasing [8].

Existing evidence suggests that physical activity (PA) in pregnancy is inversely associated with GWG [9] and BW [10]. However, it is difficult to accurately assess when and how PA impacts GWG and BW, as the effect can be influenced by a myriad of factors, including the volume, intensity, and type of PA, and the timing of exercise during pregnancy [9,11,12,13]. Most previous studies have focused on the effects of exercise during only one trimester, included only moderate or vigorous activity, or used assessment tools that have not been validated to measure the PA during pregnancy (e.g., household activity, a main activity for pregnant women, was rarely included) [9,12,13]. Moreover, most previous studies have examined the association of PA and GWG or BW without excluding the influence of energy intake [10,14], an important covariate affecting GWG and PA behavior [9,15,16]. The previous findings on PA during pregnancy are not widely applied in intervention programs due to limitations in research design, measurement, and sampling. In addition, the effect of PA during pregnancy on GWG and BW has been demonstrated to be race-dependent [3,10,14,17]. China has one of the largest populations of pregnant women in the world, but little is known about how PA during pregnancy can affect maternal and infant health in China. 

Given the limitations of previous research and the unique characteristics of Chinese pregnant women, we aim to examine the effect of PA during pregnancy in the female Chinese population by taking the total volume, intensity, type, and timing of PA (while controlling for the influence of energy intake) into consideration. To date, the recommendation for pregnant women on PA is crude and based on the recommendation for the general adult. The findings of this study may help to establish specific recommendations and interventions for pregnant women to prevent inappropriate GWG and high BW.

## 2. Material and Methods

### 2.1. Study Design and Participants

A longitudinal study was conducted. The participants were recruited through direct contact by doctors, nurses, and researchers from June 2014 to January 2016 from the Maternal and Child Health Hospital of Chengdu Wuhou in the Sichuan province of western China. A stratified sampling strategy was employed to recruit roughly equal number of participants in the first, second, and third trimesters of pregnancy (<13 weeks, 13–28 weeks, and >28 weeks, respectively). The inclusion criteria included being older than 20 years with a singleton pregnancy and having no major chronic diseases (diabetes mellitus, hypertension, heart disease, chronic renal disease, and other diseases). 

Two rounds of data collection were conducted: one during gestation and the other after delivery. The first data collection included participants’ energy intake status during pregnancy for the past 3 months, PA during pregnancy, and demographic data, such as age, education level, marital status, monthly income, and pre-pregnancy body mass index (BMI, kg/m^2^). In the second data collection, GWG and BW data were obtained from the patient medical records provided by the hospital. 

Consent was obtained from each participant. All participants were informed about the study’s purpose and procedures, and received instructions for completing the questionnaires. 

### 2.2. Assessment of PA

PA during pregnancy was assessed by using the Chinese version of the Pregnancy Physical Activity Questionnaire, which is a semiquantitative questionnaire validated for use during pregnancy (ICC = 0.77, r = 0.35) [18,19]. The original Pregnancy Physical Activity Questionnaire assesses the duration, frequency, and intensity of different types of PA during pregnancy [19]. The PA categories include: household/caregiving (13 activities), occupational (5 activities), and sports/exercise (8 activities). Activities are also classified by different intensities: light (1.5–<3.0 METs), moderate (3.0–6.0 METs), or vigorous (>6.0 METs). Total PA (total MET·hour (h)/week) (non-sedentary) was summarized by intensity category. Based on a previous study [14], total PA and each PA were divided into four quartiles with the fourth reflecting the highest level of activity. Due to the small number of participants in the vigorous activity group (*n =* 49), vigorous activity was categorized into two types (any or none). For the same reason, three categories (unemployed, low, and high) were set for the occupational activity. The low (*n =* 148) and high (*n =* 158) occupational groups were divided by the median.

Based on the international guideline, which recommends moderate-intensity exercise for at least 20–30 min per day on most or all days of the week [20] and a previous study [14], at least 7.5 MET·hour (h)/week at moderate intensity PA was considered to meet the PA recommendation.

### 2.3. Assessment of GWG and BW

We defined total GWG as the difference between weight before delivery and pre-pregnancy maternal weight. Readings of both weights were collected from the women’s medical records, which were measured by a specialist doctor one time. Total GWG was categorized into inadequate, adequate, or excessive, based on the 2009 Institute of Medicine guidelines [21]. Data about the infants included the infant BW, height, and sex, which were extracted from the medical records. Infant BW (in grams (g)) was analyzed as a continuous variable.

### 2.4. Covariate Assessment and Other Measurements

Demographic information, including age, pre-pregnancy maternal weight, height, marital status, exercise habits before pregnancy (≥3 times per week: yes or no), education (categorized as less than high school, high school graduate, or college degree or higher), and monthly household income (categorized as <4000, 4000–8000, or >8000 RMB), was obtained by a questionnaire. The semi-quantitative of food frequency questionnaire was used to evaluate energy intake status for the past 3 months for pregnant women [22].

### 2.5. Statistical Analysis

The data were analyzed by following the procedure adopted by a previous study [14]. Chi-square tests were used to examine the association of sociodemographic characteristics with GWG (inadequate, adequate, and excessive). To estimate the effects of PA volume, intensity, and type on GWG, multinomial logistic regression was used with adequate GWG as the reference group. To examine how PA volume, intensity, and type are related with BW, linear regression models were performed. The odds ratios (OR) in crude and adjusted models were calculated. PA was categorized into four levels using the cut-points of quartiles, and the lowest quartile was defined as the reference group. The factors associated with GWG (i.e., age, pre-pregnancy BMI, and energy intake) were included in adjusted models. To assess other confounding factors, we evaluated each covariate by calculating changes of the ORs for PA in the regression model. A change of >10% was used as a threshold to determine confounding factors [14]. Given the criteria, age, education, pre-pregnancy BMI, marital status, exercise habits before pregnancy, household income, energy intake, and the infant’s sex were included in multivariable models. Tests of trend were calculated by modeling PA categories as continuous linear variables (e.g., 1, 2, 3, and 4) [14]. Finally, according to the covariates, participants with and without missing information were compared to confirm whether the missing data would affect the overall results. A significance value of *p* < 0.05 was used for all analyses. SPSS version 21 (IBM Corp., Armonk, NY, USA) was used to perform all statistical analyses.

## 3. Results

We recruited 1272 pregnant women. After excluding ineligible participants, 1077 (83%) participants were included in the final analysis. The weights before delivery data were available for 599 participants (55.6%), and BW data were available for 606 participants (56.3%). There were no significant differences between the included and excluded participants in terms of age, pre-pregnancy BMI, exercise habits, education, income, weeks of gestation, employment status, or household income. There were no significant differences between the included and excluded participants in terms of age, pre-pregnancy BMI, exercise habits, education, income, weeks of gestation, employment status, household income, or energy intake (*p* > 0.05), except marital status (89.8% versus 94.1%, *p* = 0.003).

In our study, less than half of the Chinese women (43.4%) had adequate/appropriate GWGs: 27.9% had inadequate GWGs, and 28.7% had excessive GWGs, as determined by the Institute of Medicine guidelines (Table 1). The mean GWG was 14.12 ± 5.38 kg. Married women (89.8%) were significantly more likely to have adequate GWGs. Most participants (69.6%) had normal weight before pregnancy. Participants who were overweight or obese (BMI > 25 kg/m^2^) before pregnancy were significantly more likely to have excessive GWG. The mean birthweight was 3344.72 ± 443.15 g, and 55% of the infants were boys. The women who had given birth to boys were significantly more likely to have excessive GWG. More than half (67%) of participants met the international guideline for PA (≥7.5 MET·hour (h)/week in moderate intensity PA). Women in the third trimester had significantly higher median total energy expenditure (62.4 MET·h/week) than women in the first and second trimesters (52.8 and 54.9 MET·h/week, respectively; *p* < 0.01). The mean energy intake was 2035.43 ± 744.39 kcal, and there was significant difference of energy intake among the three categories of GWG (inadequate, adequate, and excessive GWG).

We conducted three multinomial logistic regressions to examine the effects of total volume of PA, PA intensity, and PA type during each of the three trimesters on GWG in the crude and adjusted models (adjusting for the energy intake and other sociodemographic covariates). The results for the first trimester are shown in Table 2. In the crude and adjusted models, compared to women in the lowest quartile of total PA, women with the highest levels of total PA did not have a significantly decreased risk of inadequate GWG (OR = 0.78, 95%; confidence interval (CI) 0.25–2.42) or excessive GWG (OR = 1.24, 95%CI: 0.34–4.55). There was no significant association between the intensity of PA (i.e., low intensity, moderate, and vigorous) or type of PA (i.e., household/caregiving, occupational, and sports/exercise) and inadequate or excessive GWG. Similarly, women who meet the PA recommendations during the first trimester did not have a significantly reduced risk of inadequate or excessive GWG compared to those who did not meet the recommendations.

Table 3 shows the results for the second trimester. Similar to the results for the first trimester, we did not observe any significant associations of meeting the PA recommendations and total PA with inadequate or excessive GWG during the second trimester. There were no significant associations between intensity or types of PA activity and GWG.

The results for the third trimester are shown in Table 4. We observed that women with the highest level of total PA had a 69% reduced risk of inadequate GWG (OR = 0.31, 95%CI: 0.10–0.91) and a 67% reduced risk of excessive GWG (OR = 0.33, 95%CI: 0.12–0.91) compared to women in the lowest quartile of total PA (approximately 23 MET·h/week) in the adjusted model (including energy intake and other sociodemographic covariates). There was no significant association between meeting the PA recommendations and inadequate or excessive GWG. In addition, there were no significant associations between intensity and the type of activity with inadequate or excessive GWG during the third trimester.

The associations between PA and BW in each trimester were examined, and the results are presented in Table 5. There were significant associations between meeting the PA recommendations (≥7.5 MET·hour (h)/week in moderate intensity PA) and BW in the second and third trimesters, but not in the first trimester. Women who met the PA recommendations (≥7.5 MET·hour (h)/week in moderate intensity PA) in the second and third trimesters had infants with significantly lower BWs compared to those who did not meet the recommendations (β = −0.15, SE = 66.33, *p* = 0.04; β = −0.20, SE = 64.54, *p* = 0.01, respectively) in the crude and adjusted models. This significant association did not change when adjusting for total PA. 

## 4. Discussion

This is the first study to comprehensively examine the effect of PA during pregnancy on GWG and BW in Chinese women by considering the volume, intensity, type, and timing of PA, while controlling for the influence of energy intake. We confirmed that the latter half of pregnancy is a critical period to reduce inappropriate GWG and macrosomia for Chinese women, and the effects of total volume of PA and intensity of PA on GWG and BW were different. Women with the highest levels of total volume of PA in the third trimester of pregnancy had a significantly reduced risk of inadequate GWG and excessive GWG compared to women in the lowest quartile of total PA. Additionally, women who met recommended moderate intensity of PA in the second and third trimesters, independent of total volume of PA, had infants with significantly lower BWs compared to those who did not meet the PA recommendations. 

The current results are in line with previous studies showing that less than half of pregnant women had appropriate GWG among Chinese women [7], and that total volume of PA only in the third trimester could reduce the risk of inadequate or excessive GWG [13,23], even adjusting the influence of energy intake. Clapp et al. indicated that the anthropometric or metabolic changes through the trimesters may have led to an association between exercise and GWG only in the third trimester, but not in the first and second trimesters. The anthropometric changes during pregnancy are likely regulated by hormonal signaling from the conceptus and represent one part of the preparative response of the first and second trimesters [23]. Metabolic changes during pregnancy include a shift toward maternal insulin resistance and fetal lipid metabolism in the third trimester, and it is possible that the significant association between PA and GWG in the third trimester is partly attributed to this change in maternal and fetal metabolism [23]. Furthermore, this association was significantly attenuated if the energy intake and other covariates (in the crude model) were not adjusted. This finding showed that the effect of total PA on inappropriate GWG in the third trimester may be certainly attributed to the overall energy expenditure on other components of energy balance. 

Unlike the previous studies that showed an impact of moderate or vigorous exercise on GWG [9,12], our findings, consistent with the Chasan-Taber et al., did not show a significant association between type or intensity of PA and inadequate or excessive GWG during the first, second, and third trimesters [14]. The discrepant findings may be related to differences in participants with normal weight and overweight/obesity or in the instrument of PA assessment. Some studies showed that the pregnancy processes differ between normal weight and overweight/obese women [24,25]. For example, most participants (69.6%) had normal weight before pregnancy in our study, in contrast to only 36% in Ehrlich et al.’s study [12]. Another possible reason is the different measures used. For example, Stube et al., assessed PA using a questionnaire modified from the Physical Activity Scale for the Elderly that was not validated to measure the PA during pregnancy. Moreover, the previous studies have not considered the influence of energy intake [9,12,14]. 

We did not find a significant association of each intensity of PA or meeting the PA recommendations with GWG at any stage of pregnancy. These findings pointed to the possibility that it is more important to focus on the total volume of PA rather than intensity of PA for controlling GWG. Moreover, the level of PA from the American College of Obstetricians and Gynecologists’ guidelines (total volume ≥ 7.5 MET·hour (h)/week in moderate intensity PA) may be insufficient to affect the GWG for Chinese women. Our previous study showed Chinese pregnant women have high total PA [26], and this study further showed that Chinese women with a higher level of total volume of PA had a lower risk of having inadequate GWG and excessive GWG, more than the volume recommended. A revised guideline may be needed for the population of Chinese women to control GWG. 

Regarding the association between PA and BW, the current study found that the infant BW was significantly lower among Chinese women who met recommended moderate intensity of PA in the second and third trimesters, even adjusting total PA volume and energy intake. This is consistent with the results of previous studies, confirming that PA recommendations are effective in preventing high infant BW or macrosomia [10,27,28]. For instance, the randomized trial by Hopkins et al. found that babies of women who participated in a bicycling program from 20 weeks of gestation until delivery were lower in weight by about 140 g than the babies of the women in the control group [28]. 

Although the mechanisms underlying how maternal PA reduces BW have not been fully clarified, one plausible hypothesis is that maternal PA influences fetal growth, in part, through endocrine regulation. Several previous studies showed that PA, particularly moderate activity in the third trimester of pregnancy, improves maternal insulin sensitivity and reduces maternal blood glucose levels, which are associated with fetal BW [29,30]. Furthermore, a randomized control trial by Hopkins et al. also found Women who participated in the moderate-intensity exercise group during the second and third trimesters significantly increased the level of leptin, which may have contributed to the reduction in offspring size [31]. These findings may partly explain why our data showed that only women who met a certain amount of moderate intensity of PA, not the light-intensity PA, in the second and third trimesters, had infants with lower BWs. In our study, even after we adjusted for the energy intake and total PA, the significance of the associations remained. This suggests that the intensity of PA during the last trimester may be important for controlling infant BW. 

Since few studies have comprehensively examined the association of PA during pregnancy with GWG and BW in Chinese women by considering the volume, intensity, type, timing of PA, and the influence of energy intake, to date, there is no consensus regarding when and how PA influences GWG and infant BW. The present findings have clinical implications. First, our data identifies the latter half of pregnancy as a critical period for controlling maternal GWG and infant BW, especially in China where there is a high proportion of women with inappropriate GWG and macrosomia. Second, the intervention of exercise program to prevent inappropriate GWG and macrosomia for Chinese women may need to consider different priorities on total volume or intensity of activity. For instance, for women with inadequate or excessive GWG, total volume of PA needs to be emphasized, and increasing light intensity PA is also suitable; but for women who have large fetuses, the intensity of PA may play a more important role. To further validate our finding, randomized controlled trials are necessary to intervene during the critical window of late trimesters and identify definite causal effects of PA on GWG and BW. 

## 5. Limitations and Strengths

Several limitations of this study are worth noting. First, the participants were recruited from only one city, which may limit the generalizability of the findings for all Chinese women. To alleviate this problem, we chose Chengdu, a large city with rural and urban regions and a population of approximately 16 million. Second, this was an observational study; therefore, causality cannot be established. We recommend randomized controlled trials to be conducted to identify definite causal effects of PA on GWG and BW. Third, the PA was measured by the participants’ self-reports in questionnaires and subjective bias could be a concern. Objective PA should measured to confirm our findings in future research.

Despite the limitations, our study has strengths in several aspects. First, the participants were assessed during all trimesters of pregnancy, which allowed us to precisely understand how the timing of PA can influence GWG and BW. Second, validated methods were used to comprehensively assess the PA activity of pregnant women, and we specifically evaluated the volume, type, and intensity of activity. Third, this study accounted for energy intake, which is associated with GWG and PA [9,16], but has been overlooked by most previous studies [10,14]. We used it in the adjusted model to remove its possibly confounding effect. Finally, we combined both survey data collected from participants and objective data from medical records, which helped to reduce method biases and provided reliable results.

## 6. Conclusions

In conclusion, the latter half of pregnancy is a critical period to reduce inappropriate GWG and macrosomia for Chinese women. Meanwhile, the total volume and the intensity of PA may be important for controlling GWG and reducing macrisomia, respectively. Future PA intervention for preventing inappropriate GWG and macrosomia may need to consider different priorities on total volume or intensity of PA and timing. Our findings support the encouragement of PA in the last trimester of pregnancy and confirm it as an important clinical strategy to control GWG and fetal BW.

## Figures and Tables

**Table 1:** Participants characteristics according to compliance with Institute of Medicine (IOM) Guidelines ^a^.

	Total		Inadequate GWG ^a^	Met GWG Guidelines	Excessive GWG	*p*-Values ^b^
	N	%	%	%	%	
Total	606		27.9	43.4	28.7	
Demographic variables						
Age(year)						0.157
<25	165	27.3	24.1	28.7	27.5	
25 < 30	301	49.7	46.4	49.6	50.9	
≥30	140	23.1	28.9	21.3	19.3	
Education						0.564
Lower than high school	113	18.6	21.1	18.4	17.1	
High school graduate	156	25.7	24.1	23.8	30.0	
College or higher degree	338	55.7	54.8	57.8	52.9	
Monthly household income (RMB)						0.574
<4000	162	26.8	28.0	24.9	27.4	
4000–8000	288	47.6	49.1	45.8	49.4	
>8000	155	25.6	23.0	29.3	23.2	
Marital status						0.018
single/divorced/separated/widowed	62	10.2	12.2	6.3	14.4	
married	544	89.8	87.8	93.7	85.6	
Pre-pregnancy BMI (kg/m^2^)						0.002
<18.5	135	22.2	15.7	29.5	18.1	
18.5–25	422	69.6	76.5	65.5	70.8	
>25	46	7.5	7.8	5.0	11.1	
Exercise habits before pregnancy						0.083
no	244	40.3	34.9	45.3	38.0	
yes	362	59.7	65.1	54.7	62.0	
infant sex						0.001
boys	330	54.5	50.0	49.4	66.9	
girls	276	45.5	50.0	50.6	33.1	

^a^ GWG, gestational weight gain; according to 2009 Institute of Medicine (IOM) guidelines. ^b^ *p*-values are from Chi square tests. Bold text indicates a statistically significant difference with a *p*-value less than 0.05.

**Table 2:** The association between first trimester physical activity and gestational weight gain (GWG) guidelines.

	Inadequate GWG ^a^				Excessive GWG ^a^			
	Crude		Adjusted ^b^		Crude		Adjusted ^b^	
	OR ^a^	(95%CI)	OR ^a^	95%CI	OR ^a^	95%CI	OR ^a^	95%CI
Total physical activity								
1st quartile	1.00	Referent	1.00	Referent	1.00	Referent	1.00	Referent
2nd quartile	0.69	(0.25, 1.92)	0.61	(0.20, 1.86)	1.54	(0.51, 4.62)	1.39	(0.40, 4.83)
3rd quartile	0.42	(0.15, 1.20)	0.33	(0.10, 1.03)	0.71	(0.22, 2.26)	0.54	(0.15, 1.98)
4th quartile	0.72	(0.26, 1.95)	0.78	(0.25, 2.42)	1.01	(0.32, 3.20)	1.24	(0.34, 4.55)
P-trend	0.34		0.37		0.64		0.84	
Physical activity by intensity								
Low intensity								
1st quartile	1.00	Referent	1.00	Referent	1.00	Referent	1.00	Referent
2nd quartile	0.98	(0.35, 2.74)	0.71	(0.23, 2.16)	0.96	(0.34, 2.76)	0.68	(0.21, 2.25)
3rd quartile	0.32	(0.10, 0.97)	0.23	(0.07, 0.77)	0.46	(0.16, 1.31)	0.38	(0.12, 1.25)
4th quartile	1.52	(0.57, 4.07)	1.70	(0.56, 5.15)	0.82	(0.27, 2.48)	1.15	(0.34, 3.94)
P-trend	0.86		0.91		0.39		0.76	
Moderate								
1st quartile	1.00	Referent	1.00	Referent	Referent	1.00	Referent
2nd quartile	0.52	(0.18, 1.49)	0.51	(0.17, 1.58)	0.61	(0.21, 1.78)	0.41	(0.12, 1.42)
3rd quartile	0.61	(0.22, 1.71)	0.57	(0.19, 1.70)	0.85	(0.30, 2.38)	0.71	(0.23, 2.18)
4th quartile	0.85	(0.32, 2.27)	0.73	(0.23, 2.26)	0.43	(0.13, 1.38)	0.31	(0.08, 1.18)
P-trend	0.84		0.57		0.27		0.18	
Vigorous								
None	1.00	Referent	1.00	Referent	1.00	Referent	1.00	Referent
Any	1.65	(0.64, 4.24)	2.12	(0.76, 5.91)	1.57	(0.58, 4.30)	1.78	(0.59, 5.37)
Physical activity by type								
sports/exercise								
1st quartile	1.00	Referent	1.00	Referent	1.00	Referent	1.00	Referent
2nd quartile	1.14	(0.36, 3.53)	1.08	(0.30, 3.87)	2.08	(0.71, 6.09)	1.72	(0.53, 5.55)
3rd quartile	2.27	(0.82, 6.32)	2.54	(0.84, 7.70)	1.60	(0.52, 4.87)	1.56	(0.48, 5.09)
4th quartile	1.88	(0.67, 5.30)	2.32	(0.76, 7.15)	1.21	(0.38, 3.84)	1.20	(0.36, 4.02)
P-trend	0.65		0.79		0.14		0.23	
Household/caregiving								
1st quartile	1.00	Referent	1.00	Referent	1.00	Referent	1.00	Referent
2nd quartile	0.80	(0.28, 2.32)	0.69	(0.22, 2.18)	0.53	(0.16, 1.77)	0.45	(0.12, 1.72)
3rd quartile	1.03	(0.40, 2.65)	0.79	(0.28, 2.28)	0.93	(0.35, 2.48)	0.81	(0.26, 2.50)
4th quartile	0.75	(0.28, 2.02)	0.69	(0.21, 2.22)	0.64	(0.23, 1.82)	0.82	(0.23, 2.89)
P-trend	0.69		0.54		0.54		0.78	
Occupation								
unemployed	1.00	Referent	1.00	Referent	1.00	Referent	1.00	Referent
Low	1.04	(0.46, 2.35)	1.10	(0.44, 2.75)	0.79	(0.33, 1.89)	0.78	(0.29, 2.11)
High	1.16	(0.44, 3.08)	1.00	(0.33, 3.06)	0.98	(0.35, 2.75)	1.03	(0.32, 3.30)
P-trend	0.78		0.97		0.86		0.96	
Met physical activity guideline								
No	1.00	Referent	1.00	Referent	1.00	Referent	1.00	Referent
Yes	1.04	(0.50, 2.17)	0.90	(0.41, 1.98)	1.00	(0.46, 2.17)	0.87	(0.37, 2.07)

^a^ ORs, Odds ratios, Compared to meeting IOM guidelines with multinomial logistic regression. ^b^ Adjusted for age, education, Pre-pregnancy BMI, marital status, exercise habits before pregnancy, household income and energy intake.

**Table 3:** The association between second trimester physical activity and gestational weight gain (GWG) guidelines.

	Inadequate GWG ^a^				Excessive GWG ^a^			
	Crude		Adjusted ^b^		Crude		Adjusted ^b^	
	OR ^a^	(95%CI)	OR ^a^	95%CI	OR ^a^	95%CI	OR ^a^	95%CI
Total physical activity								
1st quartile	1	Referent	1	Referent	1	Referent	1	Referent
2nd quartile	0.79	(0.32, 1.98)	0.66	(0.25, 1.75)	0.59	(0.23, 1.55)	0.53	(0.19, 1.51)
3rd quartile	1.02	(0.41, 2.54)	0.99	(0.38, 2.59)	0.68	(0.26, 1.79)	0.59	(0.20, 1.72)
4th quartile	0.65	(0.24, 1.76)	0.52	(0.18, 1.47)	1.19	(0.48, 2.95)	0.92	(0.34, 2.52)
P-trend	0.57		0.4		0.5		0.93	
Physical activity by intensity								
Low intensity								
1st quartile	1	Referent	1	Referent	1	Referent	1	Referent
2nd quartile	1.25	(0.49, 3.16)	1.35	(0.51, 3.62)	0.82	(0.31, 2.15)	1.02	(0.36, 2.87)
3rd quartile	0.85	(0.35, 2.07)	0.78	(0.31, 1.99)	0.61	(0.24, 1.51)	0.53	(0.20, 1.40)
4th quartile	1.1	(0.43, 2.80)	0.98	(0.37, 2.59)	1.19	(0.49, 2.92)	0.84	(0.32, 2.18)
P-trend	0.94		0.72		0.93		0.44	
Moderate								
1st quartile	1	Referent	1	Referent	1	Referent	1	Referent
2nd quartile	0.82	(0.34, 2.02)	0.79	(0.31, 2.00)	0.69	(0.26, 1.84)	0.64	(0.22, 1.81)
3rd quartile	0.88	(0.35, 2.22)	0.96	(0.36, 2.53)	1.14	(0.45, 2.92)	1.21	(0.44, 3.32)
4th quartile	0.89	(0.34, 2.33)	0.8	(0.30, 2.15)	1.58	(0.62, 4.03)	1.31	(0.49, 3.53)
P-trend	0.85		0.78		0.21		0.37	
Vigorous								
None	1	Referent	1	Referent	1	Referent	1	Referent
Any	0.9	(0.38, 2.17)	0.85	(0.34, 2.12)	2.3	(1.06, 4.99)	2.13	(0.93, 4.88)
Moderate or more								
1st quartile	1	Referent	1	Referent	1	Referent	1	Referent
2nd quartile	1.08	(0.45, 2.64)	1.04	(0.41, 2.60)	0.72	(0.27, 1.92)	0.65	(0.23, 1.86)
3rd quartile	0.74	(0.29, 1.90)	0.81	(0.30, 2.18)	1.11	(0.44, 2.79)	1.07	(0.39, 2.94)
4th quartile	1	(0.38, 2.65)	0.87	(0.32, 2.36)	1.59	(0.62, 4.09)	1.39	(0.51, 3.79)
P-trend	0.78		0.67		0.24		0.36	
Physical activity by type								
Sports/exercise								
1st quartile	1	Referent	1	Referent	1	Referent	1	Referent
2nd quartile	1.16	(0.44, 3.04)	1.08	(0.39, 3.00)	2.35	(0.92, 6.01)	2.73	(0.96, 7.75)
3rd quartile	1.03	(0.42, 2.52)	1.21	(0.47, 3.12)	0.80	(0.29, 2.19)	0.89	(0.30, 2.64)
4th quartile	1.05	(0.44, 2.54)	1.22	(0.48, 3.15)	1.27	(0.50, 3.19)	1.62	(0.57, 4.58)
P-trend	0.80		0.84		0.15		0.13	
Household/caregiving								
1st quartile	1	Referent	1	Referent	1	Referent	1	Referent
2nd quartile	0.59	(0.24, 1.48)	0.74	(0.29, 1.91)	0.43	(0.17, 1.10)	0.45	(0.16, 1.24)
3rd quartile	0.51	(0.19, 1.37)	0.5	(0.18, 1.41)	0.53	(0.20, 1.37)	0.43	(0.15, 1.23)
4th quartile	0.74	(0.28, 1.94)	0.77	(0.28, 2.10)	0.75	(0.29, 1.93)	0.61	(0.22, 1.69)
P-trend	0.54		0.5		0.69		0.37	
Occupation								
unemployed	1	Referent	1	Referent	1	Referent	1	Referent
Low	0.74	(0.33, 1.67)	0.57	(0.24, 1.34)	1.16	(0.52, 2.58)	0.95	(0.40, 2.28)
High	0.53	(0.23, 1.24)	0.41	(0.16, 1.04)	0.93	(0.41, 2.10)	0.85	(0.34, 2.13)
P-trend	0.13		0.05		0.92		0.73	
Met physical activity guideline								
No	1	Referent	1	Referent	1	Referent	1	Referent
Ye	1.27	(0.64, 2.50)	1.32	(0.65, 2.70)	1.33	(0.67, 2.63)	1.31	(0.62, 2.77)

^a^ ORs, Odds ratios, Compared to meeting IOM guidelines with multinomial logistic regression. ^b^ Adjusted for age, education, Pre-pregnancy BMI, marital status, exercise habits before pregnancy, household income and energy intake.

**Table 4:** The association between third trimester physical activity and gestational weight gain (GWG) guidelines.

	Inadequate GWG ^a^				Excessive GWG ^a^			
	Crude		Adjusted ^b^		Crude		Adjusted ^b^	
	OR^a^	(95%CI)	OR^a^	95%CI	OR^a^	95%CI	OR^a^	95%CI
Total physical activity								
1st quartile	1.00	Referent	1.00	Referent	1.00	Referent	1.00	Referent
2nd quartile	0.48	(0.19, 1.25)	0.29	(0.10, 0.84)	0.50	(0.21, 1.18)	0.41	(0.16, 1.06)
3rd quartile	0.70	(0.28, 1.79)	0.44	(0.15, 1.27)	0.58	(0.24, 1.40)	0.54	(0.20, 1.41)
4th quartile	0.61	(0.24, 1.54)	0.31	(0.10, 0.91)	0.47	(0.19, 1.15)	0.33	(0.12, 0.91)
P-trend	0.48		0.09		0.15		0.07	
Physical activity by intensity								
Low intensity								
1st quartile	1.00	Referent	1.00	Referent	1.00	Referent	1.00	Referent
2nd quartile	1.11	(0.45, 2.73)	0.82	(0.30, 2.20)	0.99	(0.42, 2.32)	0.90	(0.36, 2.28)
3rd quartile	0.94	(0.37, 2.40)	0.61	(0.21, 1.73)	1.03	(0.44, 2.43)	0.91	(0.36, 2.34)
4th quartile	0.94	(0.37, 2.40)	0.50	(0.17, 1.46)	0.86	(0.36, 2.07)	0.62	(0.23, 1.67)
P-trend	0.82		0.17		0.78		0.39	
Moderate								
1st quartile	1.00	Referent	1.00	Referent	1.00	Referent	1.00	Referent
2nd quartile	0.88	(0.35, 2.22)	0.69	(0.25, 1.90)	0.66	(0.29, 1.55)	0.52	(0.20, 1.30)
3rd quartile	0.53	(0.20, 1.43)	0.37	(0.12, 1.12)	0.54	(0.23, 1.28)	0.37	(0.14, 0.98)
4th quartile	1.06	(0.42, 2.67)	0.65	(0.22, 1.92)	0.55	(0.23, 1.36)	0.39	(0.14, 1.11)
P-trend	0.89		0.27		0.16		0.05	
Vigorous								
None	1.00	Referent	1.00	Referent	1.00	Referent	1.00	Referent
Any	1.40	(0.59, 3.34)	1.11	(0.40, 3.06)	1.99	(0.91, 4.34)	2.26	(0.95, 5.36)
Physical activity by type								
sports/exercise								
1st quartile	1.00	Referent	1.00	Referent	1.00	Referent	1.00	Referent
2nd quartile	2.00	(0.75, 5.35)	2.31	(0.76, 6.97)	1.07	(0.45, 2.54)	0.87	(0.33, 2.32)
3rd quartile	1.43	(0.52, 3.96)	1.30	(0.43, 3.95)	0.79	(0.33, 1.94)	0.72	(0.28, 1.88)
4th quartile	1.58	(0.62, 4.02)	1.84	(0.65, 5.19)	0.71	(0.31, 1.63)	0.82	(0.33, 2.00)
P-trend	0.23		0.25		0.85		0.75	
Household/caregiving								
1st quartile	1.00	Referent	1.00	Referent	1.00	Referent	1.00	Referent
2nd quartile	1.67	(0.65, 4.30)	1.07	(0.38, 3.01)	1.48	(0.63, 3.47)	1.16	(0.46, 2.92)
3rd quartile	2.21	(0.87, 5.63)	1.18	(0.40, 3.43)	1.47	(0.61, 3.54)	1.09	(0.40, 2.99)
4th quartile	1.00	(0.38, 2.60)	0.35	(0.11, 1.10)	0.83	(0.35, 1.97)	0.52	(0.19, 1.40)
P-trend	0.84		0.10		0.70		0.19	
Occupation								
unemployed	1.00	Referent	1.00	Referent	1.00	Referent	1.00	Referent
Low	0.50	(0.23, 1.09)	0.50	(0.21, 1.21)	1.01	(0.51, 1.98)	1.01	(0.48, 2.16)
High	0.60	(0.23, 1.55)	0.99	(0.35, 2.79)	0.68	(0.27, 1.69)	0.88	(0.32, 2.42
P-trend	0.13		0.57		0.50		0.88	
Met physical activity guideline								
No	1.00	Referent	1.00	Referent	1.00	Referent	1.00	Referent
Yes	0.99	(0.49, 2.00)	0.76	(0.34, 1.66)	0.74	(0.39, 1.41)	0.60	(0.29, 1.22)

^a^ ORs, Odds ratios, Compared to meeting IOM guidelines with multinomial logistic regression. ^b^ Adjusted for age, education, Pre-pregnancy BMI, marital status, exercise habits before pregnancy, household income and energy intake. Bold text indicates a statistically significant difference with a p-value less than 0.05.

**Table 5:** Beta coefficients and standard errors (SE) for the association of physical activity during pregnancy on birth weight (BW) in multinomial regression models.

		Birth Weight					
		Crude			Adjusted ^a^		
Variable		Beta	(SE)	*p*-Value	Beta	(SE)	*p*-Value
Early pregnancy							
Total physical activity							
	1st quartile	Referent			Referent		
	2nd quartile	0.08	(106.02)	0.42	0.05	(112.44)	0.61
	3rd quartile	0.05	(106.67)	0.60	0.10	(111.80)	0.32
	4th quartile	0.06	(106.02)	0.54	0.09	(112.78)	0.42
	P-trend	0.63			0.84		
Met physical activity guideline							
	No	Referent			Referent		
	Yes	−0.13	(75.97)	0.10	−0.13	(81.05)	0.14
Midpregnancy							
Total physical activity							
	1st quartile	Referent			Referent		
	2nd quartile	−0.14	(85.93)	0.10	−0.13	(88.94)	0.14
	3rd quartile	−0.19	(85.93)	0.03	−0.17	(88.97)	0.05
	4th quartile	−0.04	(86.79)	0.62	−0.05	(90.34)	0.56
	P-trend	0.52			0.56		
Met physical activity guideline							
	No	Referent			Referent		
	Yes	−0.15	(63.84)	0.03	−0.15	(66.33)	0.04
Late pregnancy							
Total physical activity							
	1st quartile	Referent			Referent		
	2nd quartile	−0.09	(82.18)	0.31	−0.09	(83.74)	0.32
	3rd quartile	−0.08	(83.29)	0.37	−0.03	(85.71)	0.74
	4th quartile	−0.09	(82.54)	0.27	−0.06	(86.31)	0.47
	P-trend	0.31			0.13		
Met physical activity guideline							
	No	Referent			Referent		
	Yes	−0.17	(62.86)	0.01	−0.20	64.53	0.01

^a^ Adjusted for age, education, Pre-pregnancy BMI, marital status, exercise habits before pregnancy, household income, energy intake and baby sex. Bold text indicates a statistically significant difference with a p-value less than 0.05.
